# Microbial Diversity Analysis of Sediment from Yeosu New Harbor of South Korea Using 16S rRNA Gene Amplicon Sequencing

**DOI:** 10.1128/MRA.01229-20

**Published:** 2021-01-14

**Authors:** Junho Lee, Ilwon Jeong, Jong-Oh Kim, Kyunghoi Kim

**Affiliations:** a Nara-ship, Pukyong National University, Busan, Republic of Korea; b Department of Ocean Engineering, Pukyong National University, Busan, Republic of Korea; c Institute of Marine Biotechnology, Pukyong National University, Busan, Republic of Korea; Indiana University, Bloomington

## Abstract

The Yeosu New Harbor in the South Korean benthic environment shows a mesotrophic environment affected by the Tsushima Current and the Seomjin River. Here, we report microbial diversity in sediments of Yeosu New Harbor based on 16S rRNA gene amplicon sequencing. The dominant bacterial phylum was *Proteobacteria* (relative abundance, 72.5 to 78.1%).

## ANNOUNCEMENT

Yeosu New Harbor is in the middle of the southern coast of South Korea. The benthic environment of Yeosu New Harbor shows a mesotrophic environment dominated by the influence of the Tsushima Current from the south and the Seomjin River from the north (1–3). Heavy metal contents of Yeosu New Harbor sediments have been severely changed by industrialization, such as the construction of a steel mill, a petrochemical complex, and a coal power plant after 1970 (2). Moreover, development of Yeosu New Harbor was accelerated due to the 2012 Yeosu World Expo (3). The benthic environment in the harbor is highly contaminated, with remarkable distinction compared to the outer harbor (4). Although sediment contamination in Yeosu New Harbor is severe, a limited environmental investigation was carried out. Contaminated sediment causes adverse effects on the pelagic environment, such as red tide and oxygen-deficient water (5, 6). Therefore, investigation of the benthic environment, including microbial diversity, is necessary (7). In the present study, 16S rRNA gene amplicon sequencing was applied to analyze Yeosu New Harbor sediment microbial diversity.

The sediments were collected once with a Peterson grab sampler (30 by 20 by 12 cm) from five stations (water depth, 12 to 15 m) at a 15-cm surface layer depth in Yeosu New Harbor in September 2019. Total DNA was extracted from 10 g of sediment using the DNeasy PowerMax soil kit (Qiagen) according to the manufacturer’s instructions. A 16S metagenomic sequencing library was prepared using a Herculase II Fusion DNA polymerase Nextera XT index kit v2 according to the manufacturer’s protocol (Illumina) with the primers Bakt_341F and Bakt_805R. The prepared library was sequenced with the Illumina Miseq platform at Macrogen, Inc. (South Korea). The raw reads shorter than 300 bp or with a low quality score (average score, <20) were removed. The number of operational taxonomic units (OTUs) was determined by *de novo* clustering of the sequences with a 97% sequence identity cutoff using QIIME software v1.8.0 with default parameters (8). The coordinates of the five stations and the summary of the sequencing data are presented in Table 1.

**TABLE 1:** Summary data description of collected samples in Yeosu New Harbor

Collection point	Coordinates	No. of raw reads	No. of OTUs
Y2	34°44.812′N, 127°45.445′E	231,384	13,547
Y3	34°45.15′N, 127°45.852′E	246,010	15,758
Y5	34°45.009′N, 127°45.516′E	239,922	15,981
Y6	34°44.861′N, 127°45.249′E	226,836	15,845
Y8	34°44.695′N, 127°45.591′E	267,968	16,003

**FIG 1:**
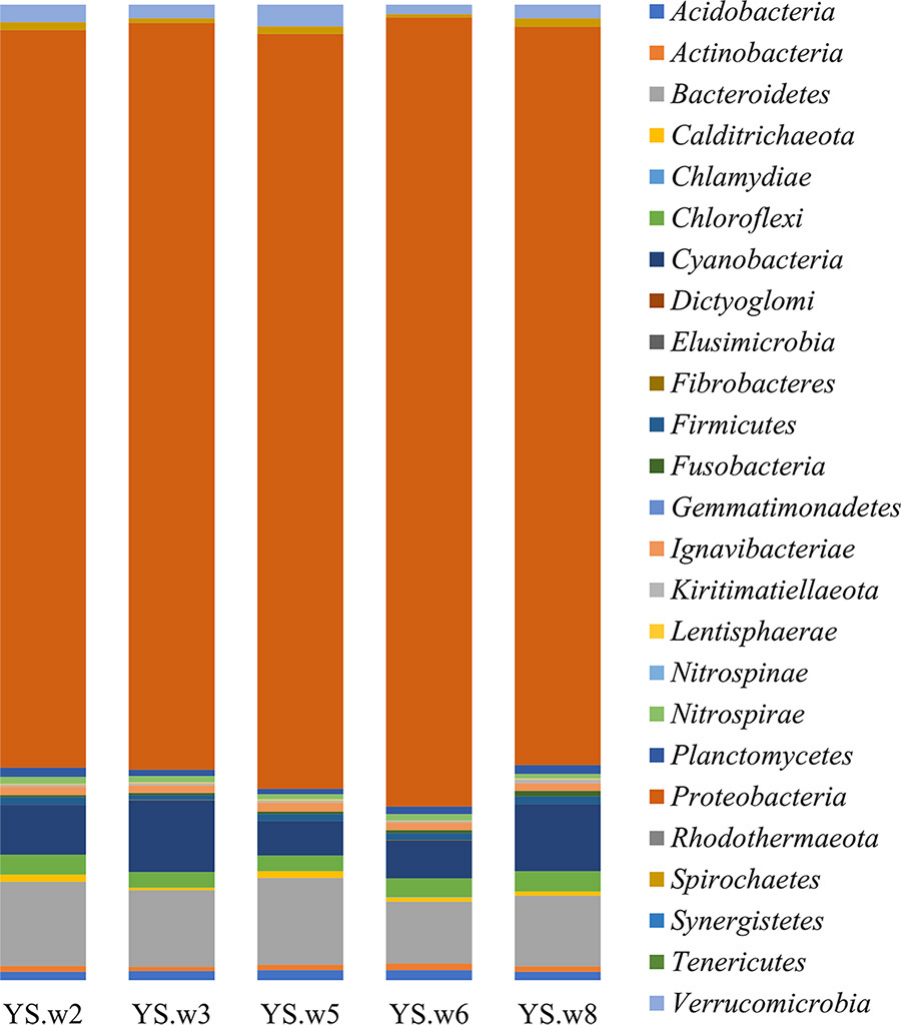
Relative abundance of the bacterial phyla in each position. Each color represents a different phylum.

Bacterial community analysis based on 16S rRNA gene analysis showed *Proteobacteria* as the predominant phylum (relative abundance, 72.5 to 78.1%), followed by *Bacteroidetes* (6.1 to 8.6%), *Cyanobacteria* (3.4 to 7.2%), *Chloroflexi* (1.5 to 2.0%), *Verrucomicrobia* (0.9 to 2.1%), *Acidobacteria* (0.8 to 1.0%), *Ignavibaceriae* (0.7 to 0.9%), *Planctomycetes* (0.6 to 0.9%), *Firmicutes* (0.5 to 0.7%), *Spirochaetes* (0.4 to 0.8%), *Nitrospirae* (0.5 to 0.6%), *Actinobacteria* (0.5 to 0.6%), *Calditrichaeota* (0.3 to 0.7%), *Fusobacteria* (0.2 to 0.5%), *Kiritimatiellaeota* (0.2 to 0.4%), and *Lentisphaerae* (0 to 0.1%) (Fig. 1). The extremely excessive abundance of *Proteobacteria* reveals the massive anthropogenic pressure in this harbor and also represents the environmental dynamics that have changed because of pollution. The results of this study are the first reporting of bacterial communities applying high-throughput DNA sequencing methods in Yeosu New Harbor and support in developing suitable policies and management tools for this harbor.

### Data availability.

The 16S rRNA gene amplicon sequences obtained from this study have been deposited in the Sequence Read Archive (SRA) of the NCBI database under the accession number PRJNA670833.
